# Challenges in Providing Counselling to MSM in Highly Stigmatized Contexts: Results of a Qualitative Study from Kenya

**DOI:** 10.1371/journal.pone.0064527

**Published:** 2013-06-07

**Authors:** Miriam Taegtmeyer, Alun Davies, Mary Mwangome, Elisabeth M. van der Elst, Susan M. Graham, Matt A. Price, Eduard J. Sanders

**Affiliations:** 1 Department of International Public Health, Liverpool School of Tropical Medicine, Liverpool, United Kingdom; 2 Centre for Geographic Medicine Research-Coast, Kenya Medical Research Institute, Kilifi, Kenya; 3 Department of Global Health, University of Washington, Seattle, Washington, United States of America; 4 Department of Medical Affairs, International AIDS Vaccine Initiative, New York, New York, United States of America; 5 Department of Epidemiology and Biostatistics, University of California San Francisco, San Francisco, California, United States of America; 6 Nuffield Department of Clinical Medicine, University of Oxford, Headington, Oxford, United Kingdom; Indiana University, United States of America

## Abstract

The role of men who have sex with men (MSM) in the African HIV epidemic is gaining recognition yet capacity to address the HIV prevention needs of this group is limited. HIV testing and counselling is not only a critical entry point for biomedical HIV prevention interventions, such as pre-exposure prophylaxis, rectal microbicides and early treatment initiation, but is also an opportunity for focused risk reduction counselling that can support individuals living in difficult circumstances. For prevention efforts to succeed, however, MSM need to access services and they will only do so if these are non-judgmental, informative, focused on their needs, and of clear benefit. This study aimed to understand Kenyan providers' attitudes towards and experiences with counselling MSM in a research clinic targeting this group for HIV prevention. We used in-depth interviews to explore values, attitudes and cognitive and social constructs of 13 counsellors and 3 clinicians providing services to MSM at this clinic. Service providers felt that despite their growing experience, more targeted training would have been helpful to improve their effectiveness in MSM-specific risk reduction counselling. They wanted greater familiarity with MSM in Kenya to better understand the root causes of MSM risk-taking (e.g., poverty, sex work, substance abuse, misconceptions about transmission, stigma, and sexual desire) and felt frustrated at the perceived intractability of some of their clients' issues. In addition, they identified training needs on how to question men about specific risk behaviours, improved strategies for negotiating risk reduction with counselling clients, and improved support supervision from senior counsellors. This paper describes the themes arising from these interviews and makes practical recommendations on training and support supervision systems for nascent MSM HIV prevention programmes in Africa.

## Introduction

Sex between men in sub-Saharan Africa has only recently gained recognition as a potential target for HIV prevention [1], [2], was the subject of a recent Lancet supplement [3] and is increasingly discussed at international conferences. Counsellors working in this region face challenges due to social stigma surrounding men who have sex with men's (MSM) behaviour and the fact that homosexual acts remain illegal in 36 African countries [1], [4], [5]. Research undertaken in Kenya and elsewhere indicates that MSM have a higher HIV prevalence than the general population in Africa [1], [6], have high rates of bisexual concurrency [7], may be active in transactional sex, and are particularly vulnerable to HIV acquisition [7]–[11]. Providing counsellors with skills to effectively provide HIV risk reduction messages tailored for MSM is an important public health target.

Current counselling, training and service provision through the voluntary counselling and testing (VCT) model in Kenya [12] have focused almost exclusively on heterosexual HIV transmission. Anal sex is rarely discussed even among heterosexual couples, and little time is given to discussion of sexual orientation or of personal values relating to homosexuality [13]. Societal attitudes regarding homosexuality and sex work sometimes conflict with counsellors' professional requirement to provide non-judgemental risk reduction counselling. This tension may provide significant stress to VCT counsellors or clinicians serving MSM. Training of Kenyan counsellors stresses the importance of supportive supervision and its role in burnout prevention, in refresher training and in tackling occasions where counsellors have felt their values were challenged [13]. There is no specific training in recognition of mental health issues.

Using the theoretical frame work of constructivism [14], we investigated attitudes towards MSM among counsellors on the Kenyan coast in one of the first MSM cohorts established in Africa [8], [9]. Constructivism is derived from the concept that reality is generated by the individual and personal experience cannot be separated from knowledge, attitudes and behaviour [15], [16]. Constructivism can be divided into cognitive and social levels. Cognitive constructivism maintains that individuals develop their own models of reality using a combination of personal experience and research-based data. Social constructivism argues that individuals are members of a community and that this shapes their models of reality [14]. Sexuality has been described as a social construct, with the meanings attached to it reflecting social and cultural values. A social constructionist approach has been applied to understanding perceptions of STIs, sexual behaviour and sexuality [17]–[20]. We used this theoretical framework to explore counsellors' attitudes and perceptions of working with MSM in Kenya and any potential impact these might have on their ability to perform risk reduction counselling for this group.

## Methods

### Ethics statement

All study participants provided written informed consent. This study received approval from the Kenya Medical Research Institute's National Ethical Review Board.

### Participants and setting

The research was conducted in June and July 2008 in a research clinic enrolling both HIV-seronegative and HIV-seropositive adults as cohort ‘volunteers’ near Mombasa, Kenya. Individuals at high risk of HIV based on a history of transactional and/or anal sex are enrolled into the cohort [8], [9], [21]. The majority of study cohort volunteers are MSM and all are offered counselling at monthly or three-monthly follow-up visits, no cohort volunteers were interviewed for this study but data on their behaviours and attitudes are reported elsewhere [9], [22], [23]. All counsellors and clinicians involved in conducting HIV-1 counselling and testing, risk assessment, screening for or treatment of sexually transmitted infections were interviewed. All of the counsellors had undertaken either the certificate in VCT or the additional higher diploma in counselling offered in Kenya. None had any training in mental health. In this paper counsellors and clinicians are referred to as ‘participants’ and cohort enrolees are referred to as ‘volunteers’. The term “participant” was selected only to distinguish from cohort volunteers, as all study participation was strictly voluntary.

### Data collection

A qualitative approach was chosen, using semi-structured, in-depth interviews in order to the elicit depth and provide detailed responses on a range of concepts [24], [25]. They were conducted in English by the first author, who was not affiliated with the site and staff. Interviews were audio-taped and transcribed within 24 hours. Participants were assured of confidentiality and written informed consent obtained. In keeping with similar studies [26], interviews were structured in such a way that information relating to homophobia was not explicitly solicited but open-ended questions were used to assess personal strengths, weaknesses, and challenges related to working with MSM. Topic guides included questions on length of experience, how they felt about working with MSM, challenges and motivators for working with MSM and how these might have changed over time and whether and how working with a group reporting illegal activity (i.e., male-male sex) has impacted their work or personal life. Specific questions about risk behaviours, behaviour change and risk reduction counselling were also asked. Participant responses were double-checked with the participant during the interview and compared with hand-written notes [27]. Findings from the interviews were incorporated into scenarios and used in a 3-day skills-building training for counsellors.

### Data analysis

Data analysis was an iterative process [28], allowing for inclusion of themes emerging from the first few interviews in the subsequent interviews and in the 3-day training. Saturation [29]was reached early in the counsellor interviews with consistent themes emerging, but interviews continued to ensure that all participants who worked on the project had a chance to have their voices included. It would have been desirable to continue to interview clinicians until saturation of data is achieved. However in this study setting, the interviews were limited to the number of clinicians working at the clinic. Despite this, on reflection, clinical staff felt that their issues had been adequately covered and there was significant overlap with themes emerging from the counsellor participants. A ‘framework’ approach was used for analysis [30], and systematically applied to sort the data. Emerging themes were sorted and coded by hand by the first author, and a selection of transcripts were re-read and coded for quality assurance purposes by an independent experienced social scientist. Analyzed data were reviewed independently by two additional social scientists and a counsellor participant to ensure that interpretations were appropriate. After analysis and training, feedback on study findings was presented to all staff involved in the research for comment in an open discussion. These discussions and reflections contributed to the validation of the data.

## Results

Three clinician participants who were providing treatment for sexually transmitted infections and general medical care and 13 counsellor participants who were providing risk reduction counselling were interviewed for this study. In total 7 women and 9 men were interviewed. Four participants self-reported as being from the MSM community in Mombasa. All 16 had first-hand experience of working with high-risk MSM for at least two years and had a combined experience of over 2,400 interactions with over 480 MSM, as well as experience with female sex workers and other populations at risk for HIV acquisition. Participants represented a variety of religious backgrounds including Muslim, Christian and agnostic. Counsellor participants were trained in the VCT model of counselling and were conversant with standard protocols. Five had a higher diploma in counselling, 8 had a certificate in counselling, and 3 were clinically qualified. Clinician participants had not received specific counselling training outside of their medical training.

### What participants knew and learned about working with MSM (the cognitive construct)

Counsellors' knowledge and experience of issues related to counselling MSM varied between the service providers who identified themselves as MSM and those who did not. Little in the standard Kenyan VCT training prepared them for the kind of counselling needs they faced and the specific challenges of risk reduction counselling in this setting. On-the-job training and peer-to-peer support was often important in learning how to provide appropriate counselling.

### Knowledge and perceptions of sexual roles amongst MSM

Participants expressed an awareness of the variety of roles played by cohort MSM. They describe ‘insertors’ (also known as ‘basha’, ‘top’ or ‘king’) and ‘receptors’ (‘shoga’, ‘bottom’ or ‘queen’). Six (6/16) of the participants reported that most MSM were taking on both roles at different times and with different partners.

Twelve participants noted that among the community at large there seemed to be a perception that to be an insertor was more socially acceptable than a receptor:

*For someone to feel comfortable that they are accepted in society they just say that I am an insertor. (non-MSM participant).*

It was suggested that this role may influence the counselling provided because some counsellors perceived that insertors would be able to ‘give up’ MSM activity or even be ‘cured’:

*It seems like insertors are held at a higher – what do I call it – you are respected if you are an insertor. It seems like counsellors probably view insertors as people who can change. ‘He is an insertor. He can change’. If he is a receptor ‘he can't change. He is already used to this habit’. (MSM participant).*

### Reported volunteer risk behaviours and triggers for high-risk behaviour

Participants reported stress when faced with reports of risk taking from the volunteers and said they faced difficulty in providing effective risk-reduction counselling. One participant describes the difficulties of counselling and challenges faced by the MSM cohort volunteers:

*As a counsellor I am like: what I am supposed to be saying? I mean how do I help in such? (non-MSM participant).*

All participants reported that MSM volunteers used condoms infrequently and they found this hard to accept as counsellors; two reported that clients had flat out refused condoms, the other 14 talked of them ‘trying but not liking’ condoms or of only using them sometimes. They described how some impoverished cohort volunteers have unprotected sex regardless of HIV status and how as counsellors they had mixed feelings, describing empathy felt by some participants towards the poor predicament of volunteer, in some ways qualifying risk behaviour and low condom use as understandable for male sex workers under pressure. This counsellor paraphrased the words of a male sex worker:

*When I have slept hungry and don't know where and when my next meal will come from and here I have somebody who would want to have anal sex with me without a condom…. I give in because I am looking forward to that meal. (MSM counsellor paraphrasing a comment from a male sex worker volunteer).*

Alcohol and drugs were described by all participants as being associated with high-risk volunteer behaviour and that when they encountered it they felt unable to work effectively with the volunteers. One counsellor describes alcohol abuse as a trigger for unsafe behaviour:

*We have discovered they mostly have sex when they are drunk… so that they don't reason. That's why most of them seroconverted. (non-MSM participant).*

Participants identified common myths among volunteers that may affect reduced condom use, including that anal sex is less risky than vaginal sex and that sex with women has a higher risk of HIV transmission since more women than men in Kenya are HIV infected. They voiced that giving information and debunking myths was a useful thing that they could do as counsellors.

Nine (9/16) participants stated that they felt hopeless when faced with volunteers who said that they preferred sex without condoms. They felt that they lacked skills to explore the reasons for this in the context of MSM relationships, and to follow through with appropriate solutions to help reduce risk taking behaviour.

Participants described feeling stressed by attitudes to condoms among the cohort volunteers and feeling that there was little they could do to impact low levels of condom use. Five (5/16) participants said that volunteers reported unprotected sex for pleasure. The remainder felt sexual risk-taking was an issue of ‘promiscuity’ (‘*being faithful for them, it cannot work*’) rather than rooted in love or desire. Themes of attraction, desire and trust as this relates to condom use were mentioned by three out of four MSM participants and by one non-MSM participant. Participants described MSM as generally unlikely to use condoms (‘*they do not like them*’ or ‘*they get more money without*’). The participants reported that as volunteer trust in their respective sexual partners increased, condom use tended to fall.

*You have been using condom but now you are good friends so you just go on without. (MSM participant).*

### Exploration of relationships, and self-esteem during counselling session

All participants reported that they rarely had time to explore wider life issues with volunteers. Participants found this challenging as the need to delve deeper frequently arose in counselling sessions. Seven participants mentioned that volunteers reported rejection by their families, feelings of isolation and having low self-esteem. In addition, stressful volunteer life events related to the inability to meet basic needs were raised. This participant, paraphrasing the words of a volunteer summed up the limitations of addressing risky behaviour without addressing the larger context of a volunteer's life:

*I imagine sometimes if I want to go to someone for counselling and they just addressed my recent sexual exploits they would not be helping me. (MSM participant).*

Participants said that low self-esteem amongst volunteers could be a factor driving risk-taking behaviour, and that stigma and shame were factors that may affect MSM self-esteem to a greater degree than in non-MSM populations. As this participant pointed out, psychological distress can lead to risk-taking behaviour:

*Could these be the issues that are putting them at risk? You don't belong anywhere. You are living a double life. … When you realize you have nothing to lose or gain you end up putting yourself at a lot of risk. When you think ‘I deserve to die’ you become someone who can take any risk… Drink [and] drive, do a lot of crazy things. (MSM participant).*

Counsellors felt that longer counselling sessions would give opportunities for deeper exploration of underlying causes, such as life circumstances and self-esteem, in order to address risk-taking behaviour during counselling sessions.

### Perceptions of the VCT model in relation to providing risk reduction counselling for MSM

Despite seeing volunteers quarterly and gaining an increased awareness of volunteer life issues and risk behaviour, all participants felt that the risk reduction counselling they provided was not adequate. Most participants felt that VCT training had equipped them well for a heterosexual HIV testing and counselling session, they often felt ill-prepared to tackle MSM-specific issues.

*We didn't go to specifics though we discussed about stigma and accepting all clients and treating them right. That was very inadequate for the kind of work I am doing. (non-MSM participant).*

*The other problem working with this group is stigmatisation and [their] feeling of being judged. ... The minute they sense a ‘stop [the MSM behaviour]’ message they will go. That is not our mandate. (non-MSM participant).*

Experienced counsellors acknowledged that sessions were more focused on probing for details of risk exposure and on telling people to use condoms than on exploring risk reduction options from the volunteers' perspectives. Most felt that coupled with providing longer counselling sessions, counselling training should focus more on counselling skills (such as reflecting, challenging, focusing, summarising and addressing loss and grief, rejection and low self-esteem.) as opposed to information giving (such as “condoms reduce transmission risk”; “lubricant reduces the risk of condoms splitting during anal sex” etc.).

*Sometimes they come and their risk is not their issue on that day. I feel that all I know is HIV and HIV related risk issues and that is all. I wish I had more knowledge and counselling skills in other areas. (non-MSM participant).*

Participants expressed frustration with their perceived lack of skill in risk reduction counselling and felt this most acutely when seroconversions happened, implying that they felt counselling had ‘failed to protect’ the volunteers.

### The influence of community values on counselling MSM (the social construct)

Social stigma and negative societal perceptions of homosexuality in Kenya often challenged participants' ability to deliver effective, non-judgemental risk reduction counselling sessions. While all participants understood the importance of not imposing one's own values during counselling sessions, the perception of homosexuality as deviant or something to be “fixed” was common.

### The impact of religious values

While all but one participant had a nominal religion nine participants described themselves as having strong religious values, both Christian and Muslim. Five participants described themselves as born again Christians. In line with their initial training, these nine participants felt that they had been successful in divorcing themselves from their religious values about homosexuality for the duration of the counselling sessions.

*Especially on my side I get satisfaction from the fact that I can divorce my religious orientation and be able to see this person as a person whose values must be respected. A person whose choices must be respected (non-MSM participant).*

However, this did not always carry over to interactions with their colleagues. All of the MSM participants described the stigma they faced from fellow counsellors:

*Stigma was there. A counsellor who was born again would tell us to stop it (MSM participant).*

*Some counsellors here they know I am an MSM. They sit me down and tell me to stop [being gay]. I usually ask myself: if they tell me that what do they tell the clients? (MSM participant).*

### Homosexuality perceived as a psychological problem

Participants described MSM, including their MSM counsellor colleagues, as ‘incongruent’. A feature of the higher diploma in counselling in Kenya is a session on ‘congruency’ which is explained as having taken a journey through one's own psychological problems. In brief, a congruent person has dealt with their issues and an incongruent person has not. An incongruent person may therefore be more likely to engage in risky behaviour than a congruent person. Three participants with the higher diploma in counselling and two without (none of whom were themselves MSM) mentioned that they did not feel MSM should become counsellors for other MSM as they were perceived as ‘incongruent’:

*How can an incongruent person help another incongruent? …. most of them they need to be helped to reach a level of accepting themselves. Before they have reached there they are already helping someone else. (non-MSM participant).*

*I feel that it is quite a challenge for a gay man to be a counsellor to another gay man because they still practice gay. (non-MSM participant).*

This highlights a perception among at least five participants who perceived that homosexuality was a psychological “problem” and that for MSM to achieve “congruency”, he must forgo sex with other men. As this MSM participant said:

*So they (non-MSM-identifying counsellors) think probably being gay is a disease. You cannot cure someone else when you already have the same disease. (MSM participant).*

Not all participants felt MSM would make poor counsellors for other MSM, however. The MSM participants and two heterosexual participants, disagreed and felt that MSM made good counsellors for MSM.

### Distinguishing sex work from sexual orientation: understanding gay relationships

Transactional sex with male clients was very common among cohort volunteers with over two thirds of participants reporting being paid for sex [31]. Perhaps as a consequence, only one (8%) of the participants explicitly distinguished sexual orientation from sex work. This lack of distinction in the majority of the participants' minds was expressed by this non-MSM participant:

*I look at a gay man as a gay man. They are the same as any other female sex worker who probably needs counselling. (non-MSM participant).*

Throughout all but one participant transcript, MSM behaviour was described as resulting from poverty (‘*they started this thing out of poverty*’) and MSM sex was regarded as transactional. The partners of volunteers were frequently described as their ‘clients’, although two of the non-MSM participants, and one MSM participant did note that some MSM did have boyfriends.

*There are some who have come forward in a relationship but their relationships are not stable. Today they will have this relationship, tomorrow they will have another. (non-MSM participant).*

Couples counselling for male couples was described as a rare event and no deliberate attempts at seeing couples were described.

### Sexual attraction in the counselling sessions

Frequent references to the issue of sexual attraction in the counselling room led to a modification of the interviews to incorporate this as a question its own right. All of the male counsellors interviewed had been propositioned at one time or another by volunteers and some confessed that they had found it quite hard to resist advances, although they knew that they were supposed to refer them to another counsellor and stated that was what they did. A number of second-hand accounts of relationships between gay counsellors and volunteers were explained. Firstly the gay counsellors stemmed from the same community that volunteers were recruited from and were previously or currently enrolled in research cohorts. Secondly they had access to records and HIV results of people they may have had sexual relationships with in the past.

*But what happened is that some clients came up with some issues. This counsellor is seducing me. Another is trying to kiss me… It was so hard. (non-MSM participant).*

*We have had issues in the counselling rooms where counsellors hit on clients or the other way around. He is nice, he gives you his number and you meet up later. (MSM participant).*

Also the counsellors lacked skill and professionalism in dealing with transference. One counsellor identified that there was a lack of real support.

*We didn't equip our counsellors to handle that – for any eventuality. We just train them to do counselling work but we don't train them on how to handle themselves professionally. (non-MSM participant).*

*The kind of supervision we have here is quite artificial. We go to supervision, I attend supervision, but we don't share those issues: for fear of course. For fear that you will be judged and for fear that your confidentiality will be compromised. (MSM participant).*

### Stigma and criminalisation

Although male-male sex and transactional sex are both illegal in Kenya, participants revealed high levels of motivation and 11 (85%) mentioned the wider public health benefits of their work as important or rewarding. They saw no conflict in their work, saying that it was not illegal to provide services to vulnerable MSM.

In stressing the importance of public health over stigma and criminalization, one participant referred to her work at a nearby antenatal clinic:

*‘As a nurse if I am in an antenatal clinic I don’t ask ‘How did you get the pregnancy? Is it legal or not?’ (non-MSM participant).*

Participants reported difficulty in gaining the trust of MSM volunteers, and volunteer recruitment efforts were initially challenging as well. Eight (8/16) participants reported that volunteers told them of being rounded up and arrested. Whilst describing themselves as ‘strong’ and ‘I am OK with it’ the four MSM participants talked of the day-to-day stresses of hiding their identity from neighbours, continually gauging people's reactions, being barred from certain places, and living ‘double lives’.

### The positive influence of peers and volunteers on value systems (changing unhelpful social and cognitive constructs)

All of the non-MSM participants commented on the way their attitudes towards MSM had changed over time as they worked with them both as peers (fellow counsellors) and as persons who needed HIV testing and counselling:

*I have changed a lot. I have to be honest – I used to not even want to work with them. Now we talk. We can go in one bus. I feel warm. These people are human beings and it is their choice. (non-MSM participant).*

*We would meet with them every day. It is like flooding. I really had to sort out my issues. Coming here really did help me…to work out my own confusions at that time. (non-MSM participant).*

They reported enjoying the relationships they had formed and shedding the stigma they once felt. A general sense of shifting values was also felt by the MSM participants, who reported that with time they experienced fewer negative comments from colleagues and felt more supported by the team.

### Training and supervision needs identified by study participants

A number of specific needs were identified and are presented in detail in Table 1. In response to their skill needs, participants felt that tailored training was required for MSM counselling to equip counsellors with skills to support condom negotiation and activities that raise self esteem. Training and supervision should also challenge homophobia and explore personal cultural traditions and assumptions. Training recommendations included development of tools to deal with self-esteem issues and dispel transmission myths common among MSM; reinforcing the importance of leaving judgemental values and homophobia at home or better yet learning why those values are harmful and ultimately shedding them altogether; and learning to distinguish between men who sell sex to other men and MSM who do not (distinguishing sex work from sexual orientation) and how to address the needs of both groups. Suggestions for supervisory support included further discussions of root causes for risky behaviour in the MSM community and to reinforce the importance of challenging negative stereotypes and fostering a safe environment for MSM (including counsellors) to be open and candid about their lives. Both recommendations touched upon improved outreach to the local professional community, including lawyers, police and community leaders.

**Table 1:** Training and supervision needs identified by study participants.

Theme	Key findings	Training needs	Supervisory support
**Inadequate skills for** **appropriate risk reduction** **counselling**	Variety of triggers: financial, drugs and alcohol, emotional, desire.	Training tailored for counselling MSM and dispelling myths	Practice skills-building in supervision sessions
	Myths (e.g. anal sex and/or sex between men is less risky)	Develop skills to exploring triggers for protected and unprotected sex	Set up exchange or counsellor mentorship programmes
		Develop job aide checklist to focus discussion and challenge client attitudes	Explore linkages to other service providers in the area
			Regular refresher training
			Information about available biomedical interventions (e.g., PreP and treatment as prevention)
**Inability to address client** **life issues**	Issues specific to MSM included: Rejection, isolation, low self esteem, leading a double life.	Provide training and observed skills practice in general counselling	Supervision sessions to explore underlying root-causes of risk behaviour
	Staff feel helpless when sero-conversions occur and ‘express frustration’ with clients	Provide skills building to prevent counselling sessions from becoming ‘stuck’ when clients say they don't want condoms.	Training to improve understanding of life issues that affect HIV prevention
		Develop activities to address low client self-esteem	
**Personal, cultural and** **religious value systems**	Challenges in maintaining a neutral attitude in the counselling process	Reinforce that homophobia has no place in counselling. Include exercises about religion and MSM in training.	Encourage counsellors to challenge their attitudes on an on-going basis.
		Explore personal and cultural traditions and assumptions.	Challenge counsellors who may over-estimate their ability to divorce their values from practice
		Learn to be aware of ‘subtle’ language.	Develop self and external assessment exercises for counsellors
		Learn to communicate support through affirming rather than ‘tolerating’ difference	
**Distinguishing sex work** **from sexual orientation**	Gay relationships are misunderstood by some staff	Develop separate HIV-test counselling protocols tailored to the needs of male sex workers and gay men who are not sex workers	Support counsellors in exploring and understanding sexual orientation, as unchallenged assumptions exacerbate stereotypes.
			Continue interaction with high risk MSM
**Unfamiliarity and lack** **of exposure**	Perception of ‘gayness’ as something that needs to be fixed.	Include training on professionalism	Tailor supervision to challenge stigmatizing values and reinforce appropriate attitudes and practice.
	Continued exposure to male sex workers and MSM clients and co-workers has improved professionalism	Use trainers who are themselves openly MSM in the training so that counsellors are not only learning on the job	Develop team building exercises to break down divisions based on sexual orientation.
			Consider peer support groups that contain a mix of gay and non-gay identifying counsellors
			Conduct training on MSM issues and create a safe space in the learning and work settings.
**Sexual attraction**	Sexual advances by both clients and counsellors	Exercises on ethics, professionalism and assertiveness to be included in training	Provide tailored support supervision
			Develop clear staff policies regarding professional behaviour
**Stigma and criminalisation**	Concern about association with MSM	Sensitization training required for local professional community (hospital, police, lawyers)	Supervisors liaise with the media and police
	Client legal problems, imprisonment, and staff harassment	Train counsellors on legal issues and dealing with conflict	

## Discussion

Our findings reveal a number of distinct themes including concerns over having inadequate skills for risk reduction counselling, and challenges in addressing client life issues. A number of counsellors in this nascent MSM programme described initial internal struggles with their personal values and unfamiliarity of working with MSM, such as concerns about being associated with an MSM project. We uncovered narratives that described the challenges of delivering effective HIV risk reduction counselling to MSM as part of standard VCT counsellor training in the face of cognitive and social constructs and we make practical recommendations on training and support supervision systems for nascent MSM HIV prevention programmes in Africa that complement what is known about current knowledge and future directions for research [32].

Since VCT counsellor training had not prepared counsellors for working with MSM, most learned “on the job” about risk-taking behaviours, life events and the triggers for risk-taking behaviour. Only the four participants from the MSM community initially had any significant understanding of MSM ‘issues’ and this engagement of MSM formed the basis of building trust and ensuring a safe environment [33]. Participants found their values shifted as they learned more about MSM through their work and through talking to their colleagues, and this clinic has now evolved into a safe space seen as a clinic of choice by many MSM in the area.

Few participants felt they were able to link strategies to prevent risk-taking behaviour with life issues, self worth and stigma. MSM may have higher rates of alcohol and drug intake [34], may feel they are not at risk of HIV or fear testing [35] and avoid services they perceive as anti-gay [36]–[38], and may avoid disclosing their sexual orientation in traditional VCT sessions [1]. While participants knew about the complex issues faced by high-risk MSM in their daily lives, very few described any alteration in their approach to standard risk-reduction planning. Similarly, while participants knew that they should be non-judgemental, our data reveal that despite changing attitudes over time, many participants' strong social constructs may have affected their ability to conduct effective risk reduction counselling.

### The impact of counselling

Providing information on HIV transmission and the risk associated with unprotected receptive anal intercourse (RAI) can lead to reduced risky behaviour among African MSM [39]–[41]. When we started work with the MSM community at the Kenyan coast HIV was thought to be a “vaginal disease” that men would contract through sex with women. We postulate that correct knowledge has increased as the result of outreach and prevention efforts. Our longitudinal cohort has allowed us to collect evidence of risk reduction during follow-up and reveal that our HIV-positive cohort participants report significantly less risky behaviour than our HIV-negative cohort participants (manuscript in preparation). It would be difficult to isolate the effect of counselling versus other components (e.g., education, HIV testing, provision of condoms and lubricants) included in the package of interventions. However, it is important to note that the level of risky sexual behaviour in our cohort and in other reported studies is still considerable. For example, in the paper by Stromdahl et al [39] only 11% of Nigerian participants reported always engaging in safe sex. In the article by Raymond et al., [41] unprotected RAI in the past 6 months (reported by 24% of participants) was associated with having had an HIV test in the past 6 months. The authors noted that “perceptions of low risk to acquire or transmit HIV infection were paradoxically associated with a higher likelihood of URAI.” In our own HIV-1 negative cohort of 449 MSM followed for various lengths of follow up time during 2005–2011, on average, one out of three MSM who reported sex with men only, and one out of 17 MSM who reported sex with both men and women acquired HIV-1 per year follow up, suggesting that risk reduction counselling alone was not effective in reducing HIV-1 acquisition in Coastal Kenya [31], and demonstrating a need for additional biomedical interventions such as pre-exposure prophylaxis and for early treatment of HIV positive individuals as a prevention measure [42], [43].

Other authors have noted the significant stigma and discrimination faced by these men [38]. We find that intermittent or on-going substance abuse, in particular, is a problem that is linked with risky behaviour (manuscript in preparation). We have also reported that HIV-positive MSM who initiate antiretroviral therapy may have lower adherence and poor response to treatment, compared to other high-risk adults, including female sex workers [44]. We suspect that the stigma, substance abuse, likely mental health issues, and other problems faced by these men can be overwhelming to our counsellors. In addition, it is quite possible that non-MSM counsellors experience stress due to continued male-male sex reported by participants (whether protected or not).

### The importance of context

There is very little published literature on the needs and experiences of counsellors working with MSM in Africa and the cultural and political dimensions of HIV/AIDS are often low priority [3]. What is known is that while the VCT model is appropriate for the client-initiated testing that it was designed for, most VCT training in Africa does not address MSM issues [13], [45]. MSM groups are increasingly acting as advocates and as service providers [46] and are well placed to face the challenges of HIV in their communities allowing solutions that are generated to be contextually appropriate to Africa [33], [47] and ultimately allowing integration into general counselling services.

### Learning from programmes with other marginalised groups

HIV testing and counselling programmes with other marginalised groups, including female sex workers (FSW), also show limited or unsustained effectiveness of behavioural interventions. Lessons learned may be appropriate for MSM counselling, although female sex work is less stigmatized than male-male sex in most African settings [48], [49]. Although many of these same stressful elements (e.g., poverty, stigmatization) comprise the challenges in providing effective counselling for all marginalized groups, the negative social and cognitive stereotypes surrounding MSM introduced additional challenges in delivering efficacious counselling messages [36]. MSM-specific vulnerabilities may include psychological and cognitive factors including low self-esteem, being at increased risk of depression, self-stigmatization and being prone to blackmail and isolation [50], [51]. Also lack of knowledge about sexuality and misconceptions how to prevent transmission (as mentioned above) are different for MSM than for FSW. Sexual intercourse more often takes place in places that are more challenging for providing prevention services (parks, beaches, etc.) and in an environment of criminalization [52].

### Training and supervisory needs

The need for a formal, validated training system to better prepare counsellors to tackle the complex set of issues specific to MSM-counselling was an important outcome identified in this work and has in part led to the development of standardized tools for this purpose [53]. Training must therefore define and assess competence in the context of MSM counselling and help participants to know more about MSM (enhancing the cognitive) and recognise the impact of value systems. The limited utility of non-standardized, non-validated “on the job” training while working with MSM highlight the need for specific VCT training modules on the MSM behaviours to avoid homophobic language (both verbal and non-verbal) and to embrace affirmation as opposed to ‘tolerance’[26]. Using our constructivist framework we argue that training and support supervision can influence the cognitive and social constructs of health workers, nurturing more positive attitudes towards MSM based on a better understanding of sexuality. To this end, context-specific training materials covering the areas set out in Table 1 and newer developments around pre-exposure prophylaxis and treatment as prevention [54] are beginning to be developed and adapted [53], and have been made freely accessible on the internet (www.marps-africa.org) [30].

In the absence of formal training, the impact of counsellors' personal values (cognitive constructs) and the values of their peers and the community (social constructs) weighed heavily on counsellors' ability to conduct effective risk-reduction counselling. Better support systems and confidential supervisory counselling could assist counsellors to develop expertise and deal with the issues of personal values coming into conflict with their clients' behaviour, sexual attraction [55], professional relationships.

### Limitations

One limitation of the study was that responses may have been influenced by knowledge of the interviewer's experience working with MSM and her role in initiating VCT counsellor training in Kenya [56]. Conversely, the fact that she was an outsider to the research project may have enabled more critical and frank views. Additionally, since the participants were working in a research setting, included counsellors drawn from the local MSM community, and had over two years experience working with MSM, our study population may be significantly different from traditional VCT counsellors in Africa, where additional support and mentorship may be required. It may be unwise, therefore, to generalize these findings to other VCT counsellors who need skills to serve MSM as well, but are unlikely to counsel MSM exclusively.

## Conclusion

For African MSM early and expanded access to non-judgemental HIV testing and counselling is critical for prevention efforts. Those who test positive will then be able to access early treatment initiation, lowering onward transmission risk, and those who test negative can then access focused risk reduction counselling and support. Our findings reveal the need for a tailored training and supervisory approach that is embedded in the specific contexts of African MSM. Through an analysis of themes emerging from qualitative interviews with experienced counsellors and clinicians in Kenya this paper raises specific challenges to the status quo and shows that specific materials and training are required. It sets out areas of training, supervision and support that can guide programmes setting out to expand HIV testing and counselling programmes for MSM in similar contexts.
